# Predicting COVID-19 progression from diagnosis to recovery or death linking primary care and hospital records in Castilla y León (Spain)

**DOI:** 10.1371/journal.pone.0257613

**Published:** 2021-09-20

**Authors:** Pedro C. Álvarez-Esteban, Eustasio del Barrio, Oscar M. Rueda, Cristina Rueda

**Affiliations:** 1 Department of Statistics and Operations Research, Universidad de Valladolid, Valladolid, Spain; 2 MRC-Biostatistics Unit, University of Cambridge, Cambridge, United Kingdom; Ohio State University Wexner Medical Center Department of Surgery, UNITED STATES

## Abstract

This paper analyses COVID-19 patients’ dynamics during the first wave in the region of Castilla y León (Spain) with around 2.4 million inhabitants using multi-state competing risk survival models. From the date registered as the start of the clinical process, it is assumed that a patient can progress through three intermediate states until reaching an absorbing state of recovery or death. Demographic characteristics, epidemiological factors such as the time of infection and previous vaccinations, clinical history, complications during the course of the disease and drug therapy for hospitalised patients are considered as candidate predictors. Regarding risk factors associated with mortality and severity, consistent results with many other studies have been found, such as older age, being male, and chronic diseases. Specifically, the hospitalisation (death) rate for those over 69 is 27.2% (19.8%) versus 5.3% (0.7%) for those under 70, and for males is 14.5%(7%) versus 8.3%(4.6%)for females. Among patients with chronic diseases the highest rates of hospitalisation are 26.1% for diabetes and 26.3% for kidney disease, while the highest death rate is 21.9% for cerebrovascular disease. Moreover, specific predictors for different transitions are given, and estimates of the probability of recovery and death for each patient are provided by the model. Some interesting results obtained are that for patients infected at the end of the period the hazard of transition from hospitalisation to ICU is significatively lower (*p* < 0.001) and the hazard of transition from hospitalisation to recovery is higher (*p* < 0.001). For patients previously vaccinated against pneumococcus the hazard of transition to recovery is higher (*p* < 0.001). Finally, internal validation and calibration of the model are also performed.

## Introduction

Since the beginning of the pandemic, there has been a great interest in developing computational models that can accurately predict disease progression in COVID-19 patients. There are many papers in the literature dealing with predictive models for COVID-19 mortality or severity. Many predictors have been identified universally and considered as risk factors, such as older age, being male, and clinical conditions such as diabetes, obesity, cancer, respiratory diseases, heart, kidney, liver, and neurological disorders, among others. However, the scope of much of these studies is limited to patients with specific characteristics or a severe diagnosis, and very few deal with multistate survival models with two absorbing states, disease related death and discharge alive from primary care or hospitalisation. They are two competing events and patients should not be censored at the time of discharge, as these events are informative and they are the key for a fair understanding of the processes involved in the disease. Furthermore, they need to be considered in order to derive unbiased risk estimators. The three main keywords of the study, Multistate, Cox Model, and COVID-19, have been used to search for related contributions, and more than 1,000 results have been obtained. It is out of the scope of the paper to make a complete revision of the bibliography, so we will mention here some of the studies that are close to ours, such as [1–11]. Compared with this study, any of them have limitations. We refer to the Discusssion section below for further details.

The present study analyses a cohort of 73,180 patients diagnosed between February and May 2020 with a clinical or virological diagnosis of COVID-19 disease and tracks their progression from the date of diagnosis to recovery or death. This cohort corresponds to all the identified infected patients during the first wave of the pandemic in Castilla y León, in Spain. Castilla y León was one of the country’s regions with the highest COVID-19 incidence and mortality rates during the period under study [12]. The original data included more than four million primary care records. This massive dataset was originated from different sources, including public hospital databases that contained complementary information on epidemiological and clinical history on the patients from hospital admission to discharge, as well as database records with results on the COVID-19 tests which were used as a criteria for patient inclusion. All these datasets were meticulously curated through a preprocessing stage in order to define the study cohort and to select epidemiological and clinical variables of interest.

**Fig 1:**
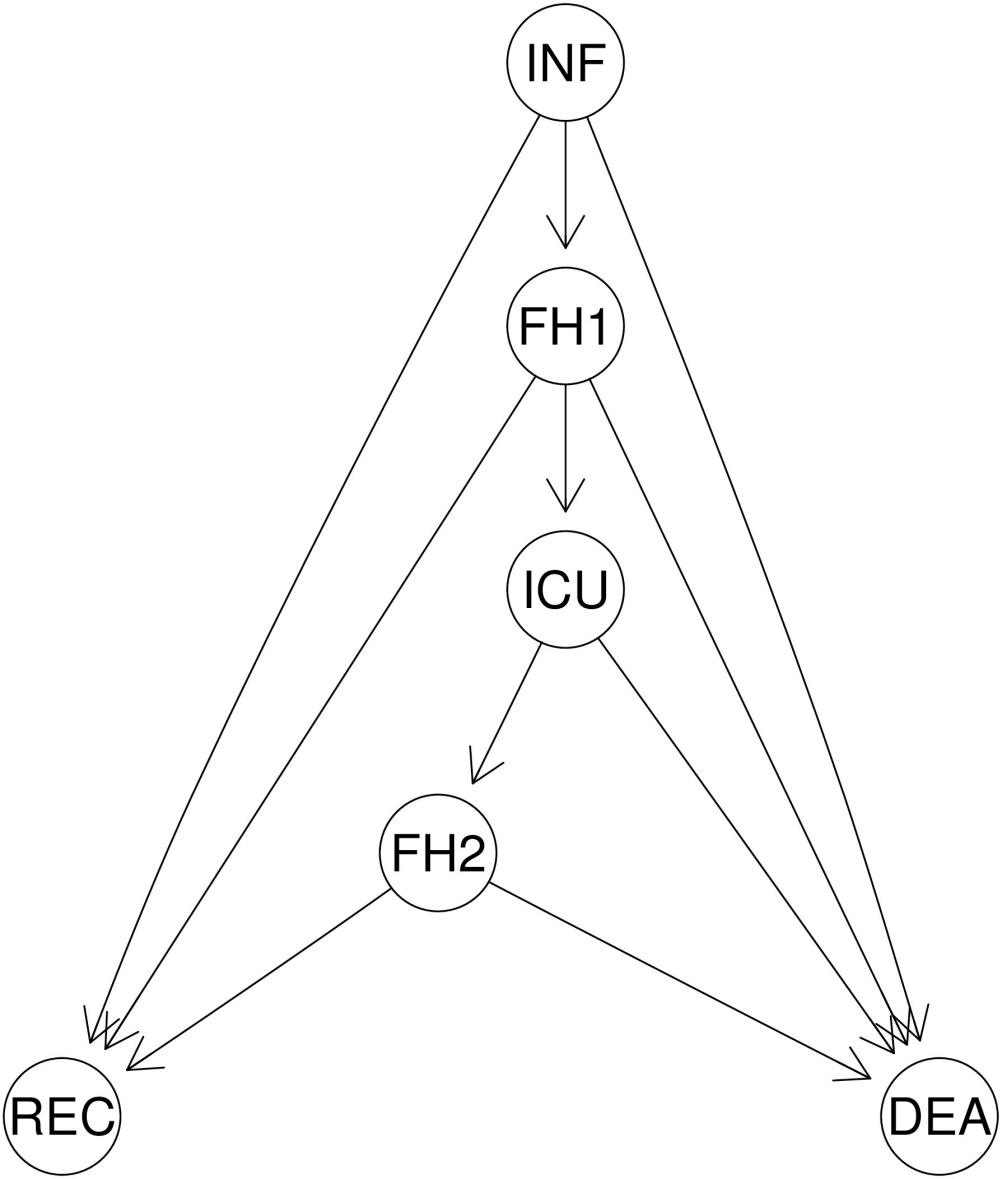
States (INF: Infected, FH1: Floor hospitalisation, ICU: Intensive care unit hospitalisation, FH2: Floor hospitalisation after ICU, DEA: Death due to infection, REC: Recovery from infection) and transitions for the multistate model.

The final dataset contained information on the clinical history of each patient, including dates of hospitalisation, intensive care and discharge or death. The set of covariables contained basic information, such as age or sex, information on previous pathologies and vaccines, pathologies acquired after the COVID-19 diagnosis and details of treatment. The dynamics of the infection process is illustrated in Fig 1. A patient enters into the initial state the date when the infection process is first recorded (INF), followed in severe cases with Floor Hospitalisation (FH1), and, in critical cases, also followed by a stay in an ICU (Intensive Care Unit) plus a second FH (FH2) stay. From all of these states, transitions to two absorbing or end states are also considered. These two events are death due to COVID-19 infection (DEA) or recovery (REC).

The analysis of this data provides estimates of rates of hospitalisation, UCI admission, REC, or DEA stratified by epidemiological and clinical variables. Besides, as the dates of entry and exit of the different states are known, estimates of the patient’s length-of-stay in the hospital or the ICU can also be derived. Inspired in a model designed to predict breast cancer progression [13], we have derived a multistate model where transitions are modelled using Cox regression [14]. This model describes how patients progress between states and provides unbiased estimates of the risk of recovery and death (REC and DEA) taking into account censoring and competing risks. It also provides estimates of transition hazards over time and how are these affected by the different covariates considered.

## Methods

This is an observational cohort retrospective study. The population under study is the inhabitants of Castilla y León (Spain), with a total population of around 2,4 million people divided into nine provinces. The study was approved by the Institutional Review Board of the Regional Health Service of Castilla y León (SACyL), which waived the requirement for informed consent according to the type of study. The study began on March 1^st^, 2020, and ended on May 31^st^, 2020 and it links primary care (PC) with public hospitals’ electronic records. The analysis describes the progression of the patients between different clinical states until REC or DEA. The progression of patients in the study continued to be observed until July 13^th^. The endpoint of a given patient was considered censored if at the end of the study there was no information about recovery or death.

The dataset comprises various tables with information on dates of diagnosis, hospital and ICU admission, discharge, recovery, or death. Besides, data include demographic characteristics such as age, sex or province, history of clinical processes and vaccination, results of PCR test and antibody tests, and drug therapies during hospital stay.

A total of 149,832 patients were selected as suspected of being infected with COVID-19 during the period considered in the study. Out of those, 2,822 had been obtained directly from hospital admission records between March 1^st^ and May 31^st^ with a registered clinical process related to the SARS-COV-2 coronavirus (including those where the word COVID-19 was present in the record, even if the COVID-19 test was negative) and 147,010 had entered the study through PC. Among the PC group, 69,040 had a clinical process of *SARS-COV-2 coronavirus disease*, 2,715 with *SARS-COV-2 coronavirus pneumonia* and the rest presented a positive test result. Additionally, the PC database provided information on clinical historic data and the process initiation date for all those patients who were not admitted directly to the hospital, while the hospital records provided information on dates of hospital and ICU admission, discharge and death but also about clinical complications and pharmacological prescriptions during the hospital stay. The final database merged the information from these two sources and from other databases that included demographical variables, previous vaccinations, pharmacological prescriptions and test results for COVID-19.

A challenging stage of careful curation and preprocessing was carried out in order to detect and correct as many inconsistencies and errors as possible. One particularly difficult aspect was the identification of COVID-19 positive patients. The analysis of antigen or antibody detection tests revealed no definitive results for these patients. A total of 237,409 tests had been done on 110,631 individuals; out of them 132,998 were antibody tests and 104,511 were PCRs, and only 31,538 had tested positive in either of the tests (only 17,142 of them by PCR).

Only those patients that had received a definite clinical or virological diagnosis of COVID-19 disease by a physician were finally selected for the analysis. As a result of this criteria, 75,124 patients from PC with *Exposure to SARS-COV-2* diagnosis and no positive test result were eliminated. At the same time, only 1,294 out of the 2,822 from hospital admission with a clear diagnosis of suspicion of COVID-19 were selected. Additionally, some patients were discharged and subsequently admitted to the hospital a second time, while some other had been transferred from hospitals. In these cases, the date of the first admission was considered as the date of entry and the date of the last stay was taken as the date of discharge. In total, 73,180 patients were included in the study.

Different predictors were considered in the study. Basic characteristics such as age, gender, province of residence, calendar month of infection and previous pneumococcal or flu vaccination were included. Clinical data about co-morbidities were summarised in 16 variables defined using previous knowledge and identifying the most common features present in the subset of patients who had died under 65 years of age. Furthermore, variables related to complications and drug therapy (only if applied to at least 30 patients) during hospital stay were also considered as candidate predictors.

Patients with missing age or sex information were excluded (0.1%). No missing values were registered in previous vaccination or other predictors.

As mentioned, the dates of entry and exit of the different states were known or considered censored. In particular, the patients who were still under study on July 13^th^ were considered censored observations. The censoring rate was 25.5% but only 0.6% in the subset of hospitalised patients, meaning that most of the censored observations probably ended up in recovery. Additionally, patients who died after being discharged from the hospital were considered censored when the time from discharge to death was higher than three days. COVID-19 death was assumed otherwise.

A Cox proportional hazard model was used to identify the factors related to the risk of transitioning from each pair of states [15]. Model selection was performed using the following procedure: first a series of simple Cox models were fit using one factor at a time. Only those with a p-value smaller than 0.01 were selected. Then, a multiple Cox model with all these variables was fit and those coefficients with a p-value smaller than 0.01 were kept. The model was refit with the definite set of variables to obtain the final set of coefficients.

Estimated hazard ratios were obtained for each covariable and predicted transition probability functions were computed for patients of interest. The expected length of stay in different states was also computed using the corresponding function in the mstate package [16]. The proportional hazards assumption was tested using the survival package function cox.zph() [17] and inspected visually using smoothed scaled Schoenfield residual plots versus time [17]. The model was internally validated and calibrated using bootstrap following the procedure described in [18, 19]. The following statistics of predictive ability were computed: (1) Somers’ Dxy rank correlation, which is 2(c-index-0.5); (2) Nagelkerke’s R2, the square root of the proportion of log-likelihood explained by the model to the log-likelihood that could be explained by a “perfect” model, adjusted with a penalty for model complexity; (3) the slope shrinkage, a measure of how much the estimates are affected by outliers; and (4) the discrimination index D, derived from the log-likelihood at the shrunken linear predictor. The optimism in each of these statistics was quantified.

We also checked model calibration, using the procedure also described in [18, 19], consisting of (1) interpolation of the hazard function using splines on all the cases as a general function of the predictor variables and time; (2) computation of the predicted values for a given time (15, 30 or 60 days); (3) computation of the differences between observed and predicted; and (4) using 75 bootstrap datasets, computation of the optimism in those differences.

All analysis were conducted using R and the packages survival [17] and mstate [16].

## Results

### Epidemiological and clinical characteristics

Our study cohort was composed of 73,180 patients, with 42,299(57.8%) females. This latter percentage was significantly higher than the 50.8%, which was the percentage of females in Castilla y León at the moment of the study. The mean age of the COVID-19 population was 54.7 years (median, 54 years), with 50% of individuals between 40 and 71 years of age. The distribution of patients in each of the the nine provinces, Ávila, Burgos, León, Palencia, Salamanca, Segovia, Soria, Valladolid and Zamora was 7.7%, 14.4%, 15.6%, 5.6%, 16.3%, 11%, 5.8%, 18.5% and 4.9% respectively. The percentages of the total population for the respective provinces being 6.6%, 14.9%, 19.2%, 6.7%, 13.8%, 6.4%, 3.7%, 21.7% and 7.2%, which also reflect an unequal geographical incidence rate, being the provinces of Segovia and Soria, which are also closest to Madrid, the ones where the incidence rate was higher. These figures are in line with what is already known, and supported, for example, by the National Study of seroprevalence ENE-Covid (see [12]; or the Spanish Health Department website https://www.mscbs.gob.es/ciudadanos/ene-covid/home.htm).

Regarding hospital admissions, 65,171 individuals (89.1%) did not required hospitalisation, 7,465 (10.2%) were hospitalised but did not enter the ICU at any time point and 544 (0.7%) were hospitalised and entered the ICU. Finally, we counted a total of 3,843 (5,3%) deaths for COVID-19 from March 1^st^ until May 31^st^. Comparing these figures with the reported numbers by the INE (National Statistics Institute of Spain) for Castilla y León during the period considered, there were 2,995 COVID-19-related deaths and 1,702 deaths with an unidentified cause but suspicious of being COVID-19-related.

The analysis included data from all the regions. Estimates of probabilities of INF, FH1, ICU, REC and DEA, stratified by patient characteristics are shown in Table 1. As expected, these numbers show an increased risk in men compared to females and a monotonically increasing risk of hospitalisation and risk of death with age. Table 2 shows the distribution of previous and posterior pathologies in each state, while Table 3 shows the treatments prescribed to the patients. We note that only those drugs administered to at least 30 patients were considered in the multistate model, in order to remove possible spurious effects. A descriptive sub-analysis of pairwise comorbities and pairwise drug therapies, by state (including only those with frequency of at least 100 hospitalised patients), is shown in S1 and S2 Tables, respectively.

**Table 1:** Distribution of patients by demographic characteristics and states.

	INF		FH1		ICU		DEA		REC	
	n	%	n	%	n	%	n	%	n	%
GENDER										
M	30,834	100.0	4,474	14.5	402	1.3	2,166	7.0	21,092	68.4
F	42305	100.0	3,494	8.3	136	0.3	1,947	4.6	29,117	68.8
Missing	41	100.0	41	100.0	6	14.6	7	17.1	34	82.9
AGE										
0–9	3,243	100.0	38	1.2	1	0.0	0	0.0	2,476	76.3
10–19	2,321	100.0	26	1.1	0	0.0	0	0.0	1,579	68.0
20–29	5,072	100.0	51	1.0	6	0.1	2	0.0	3,663	72.2
30–39	8,688	100.0	159	1.8	19	0.2	3	0.0	6,238	71.8
40–49	12,654	100.0	467	3.7	46	0.4	27	0.2	9,022	71.3
50–59	13,294	100.0	856	6.4	87	0.7	90	0.7	9,546	71.8
60–69	9,128	100.0	1,276	14.0	181	2.0	240	2.6	6,544	71.7
70–79	6,333	100.0	1,731	27.3	170	2.7	668	10.5	4,324	68.3
80–89	7,785	100.0	2,361	30.3	24	0.3	1,720	22.1	4,476	57.5
90 +	4,606	100.0	1,003	21.8	4	0.1	1,323	29.6	2,328	50.5
Missing	56	100.0	41	73.2	6	10.7	7	12.5	47	83.9
PROVINCE										
Ávila	5,659	100.0	665	11.8	33	0.6	352	6.2	3,794	67.0
Burgos	10,543	100.0	858	8.1	77	0.7	430	4.1	7,418	70.4
León	11,439	100.0	1,446	12.6	66	0.6	695	6.1	6,688	58.5
Palencia	4,220	100.0	544	12.9	33	0.8	202	4.8	2,403	56.9
Salamanca	11,928	100.0	990	8.3	86	0.7	727	6.1	8,425	70.6
Segovia	8,052	100.0	805	10.0	60	0.7	553	6.9	6,251	77.6
Soria	4,251	100.0	404	9.5	32	0.8	271	6.4	3,390	79.7
Valladolid	13,517	100.0	1,769	13.1	126	0.9	701	5.2	9,621	71.2
Zamora	3,571	100.0	528	14.8	31	0.9	189	5.3	2,253	63.1
ALL	73180	100.0	8009	10.94	544	0.74	4120	5.63	50243	68.87

**Table 2:** Distribution of patients by comorbidities, complications and states.

	INF		FH1		ICU		DEA		REC	
	n	%	n	%	n	%	n	%	n	%
Pneumococcal vaccination	2,030	100.0	276	13.6	25	1.2	76	3.7	1,541	75.9
Flu2017 vaccination	14,291	100.0	3,251	22.7	168	1.2	2,084	14.6	9,274	64.9
Flu2018 vacinnation	15,865	100.0	3,552	22.4	172	1.1	2,334	14.7	10,246	64.6
Flu2019 vaccination	18,252	100.0	3,952	21.7	196	1.1	2,640	14.5	11,663	63.9
Tobacco disorder	7,503	100.0	738	9.8	73	1.0	224	3.0	5,414	72.2
Cancer	6,950	100.0	1,653	23.8	78	1.1	1,040	15.0	4,445	64.0
Psyquiatric disorder	26,407	100.0	3,561	13.5	196	0.7	2,217	8.4	17.662	66.9
Diabetes	7,098	100.0	1,851	26.1	124	1.7	1,170	16.5	4.472	63.0
Hypercolesterol	19,895	100.0	3,461	17.4	253	1.3	1,798	9.0	13.432	67.5
Hypertension	19,304	100.0	4,222	21.9	261	1.4	2,598	13.5	12,400	64.2
Hypothyroidism	7,312	100.0	821	11.2	49	0.7	438	6.0	5,071	69.4
Cardiovascular disease	11,932	100.0	2,871	24.1	138	1.2	2,005	16.8	7,499	62.8
Obesity	8,692	100.0	1,475	17.0	147	1.7	666	7.7	6,007	69.1
Respiratory disease	33,692	100.0	4,548	13.5	283	0.8	2,529	7.5	22,926	68.0
Neurological disease	5,069	100.0	1,062	21.0	29	0.6	943	18.6	2,995	59.1
Hematological disorder	14,683	100.0	1,966	13.4	88	0.6	1,286	8.8	9,888	67.3
Liver disease	2,165	100.0	380	17.6	39	1.8	136	6.3	1,517	70.1
Kidney disease	4,003	100.0	1,054	26.3	38	0.9	792	19.8	2,412	60.3
Cerebrovascular disease	1,350	100.0	332	24.6	14	1.0	290	21.5	778	57.6
Other chronic disease	16,510	100.0	3,305	20.0	147	0.9	2,246	13.6	10,606	64.2
ALL	73,180	100.0	8,009	10.9	544	0.7	4,120	5.6	50,243	68.7
Pneumonia			4,227	100.0	333	7.9	1,065	25.2	3,142	74.3
Bilateral Pneumonia			1,375	100.0	151	10.8	398	28.9	970	70.5
Sepsic shock			134	100.0	11	7.3	67	50.0	66	49.3
Respiratory failure			590	100.0	66	10.4	209	35.4	376	63.7
Heart failure			184	100.0	8	3.3	51	27.7	133	72.3
Tromboembolism event			187	100.0	26	8.1	11	5.9	175	93.6
Pleuralpericardial effusion			54	100.0	2	3.0	17	31.5	37	68.5
ALL			8,009	100.0	544	6.8	2,260	28.2	5,701	71.2

**Table 3:** Distribution of patients by drug therapies and states.

	FH1		ICU		DEA		REC	
	n	%	n	%	n	%	n	%
METHYLPREDNISOLONE	3,000	100.0	228	7.6	987	32.9	1,994	66.5
PREDNISONE	1,261	100.0	82	6.5	264	20.9	984	78.0
CEFOTAXIME	25	100.0	5	20.0	7	28.0	18	72.0
CEFTRIAXONE	4,635	100.0	310	6.7	1,316	28.4	3,304	71.3
CEFDITOREN	550	100.0	25	4.5	103	18.7	445	80.9
CLARITHROMYCIN	203	100.0	22	10.8	47	23.2	155	76.4
AZITHROMYCIN	5,439	100.0	362	6.7	1,366	25.1	4,408	74.4
LEVOFLOXACIN	1,920	100.0	131	6.8	642	33.4	1,263	65.8
MOXIFLOXACIN	159	100.0	14	8.8	30	18.9	127	79.9
TEICOPLANININ	101	100.0	29	28.7	41	40.6	59	58.4
LOPINAVIR AND RITONAVIR	3,054	100.0	333	10.9	745	24.4	2,293	75.1
INTERFERON BETA-1B	406	100.0	127	31.3	136	33.5	267	65.8
BARICITINIB	26	100.0	3	11.5	7	26.9	17	65.4
ANAKINRA	101	100.0	36	35.6	39	38.6	58	57.4
TOCILIZUMAB	681	100.0	251	36.9	153	22.5	516	75.8
SILTUXIMAB	10	100.0	5	50.0	6	60.0	4	40.0
CHLOROQUINE	436	100.0	56	12.8	130	29.8	303	69.5
HYDROXYCHLOROQUINE	4,564	100.0	366	8.0	1,126	24.7	3,420	74.9
ALL	8,009	100.0	544	6.8	2,260	28.2	5,701	71.2

### Multistate Cox model

**Fig 2:**
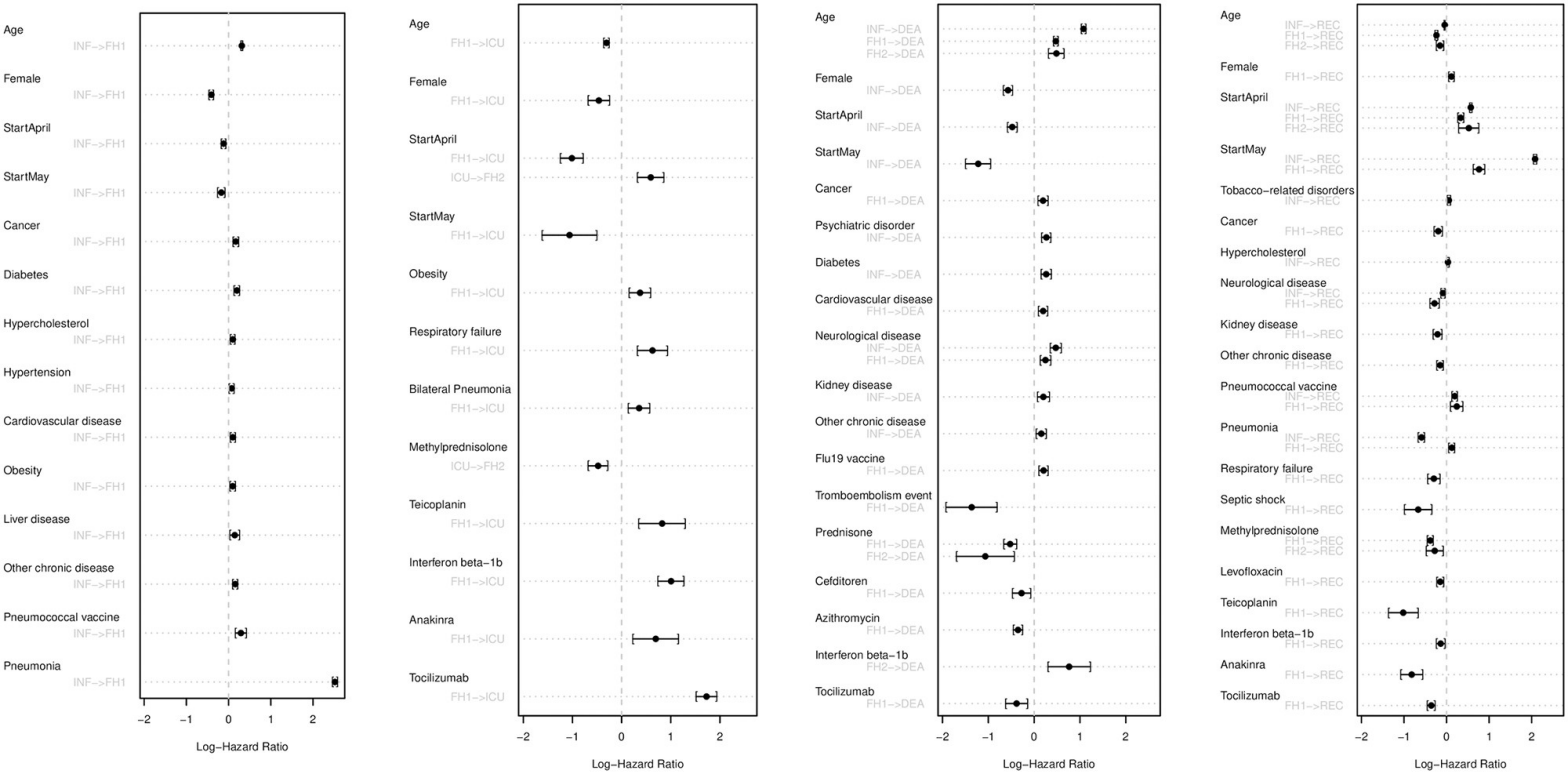
Confidence intervals for the log-hazards of each covariable split by transitions. From left to right, each panel shows parameters associated to the hazard to transition to hospitalisation, parameters associated to intensive care admission, parameters relevant to death and related to recovery.

The model represented in Fig 1 was fit to the data. This model used a clock-forward scale (keeping the time since entry throughout the process) and included 32 variables with different effects in different transitions, for a total of 78 parameters. The hazard rates for each of them are displayed in Fig 2 (numerical coefficients and p-values can be found in S3 Table).

As the figure shows, the model reflects very well the preliminary analyses performed on the cohort. From left to right, the first panel shows that age and being male increase the risk of hospitalisation. There is also a clear effect of fewer hospitalisations during April and May compared to the reference (Feb/March). There are also several previous pathologies that increase the risk of need of hospitalisation, in particular pneumonia. In terms of ICU risk, the second panel shows important factors both before and after infection, such as obesity, bilateral pneumonia or respiratory failure. The third panel shows similar effects associated to the risk of death and the last panel shows association with recovery. Our cohort is observational and not big enough in order to make statements about drug response. In particular, the results in the second panel need to be interpreted with caution, as most severe patients received specific drugs, which will be reflected as higher hazard rates of transition into ICU. However, the third panel shows that drugs such as Prednisone, Cefditoren, Azithromycin and Tocilizumab were particularly effective in our cohort.

**Fig 3:**
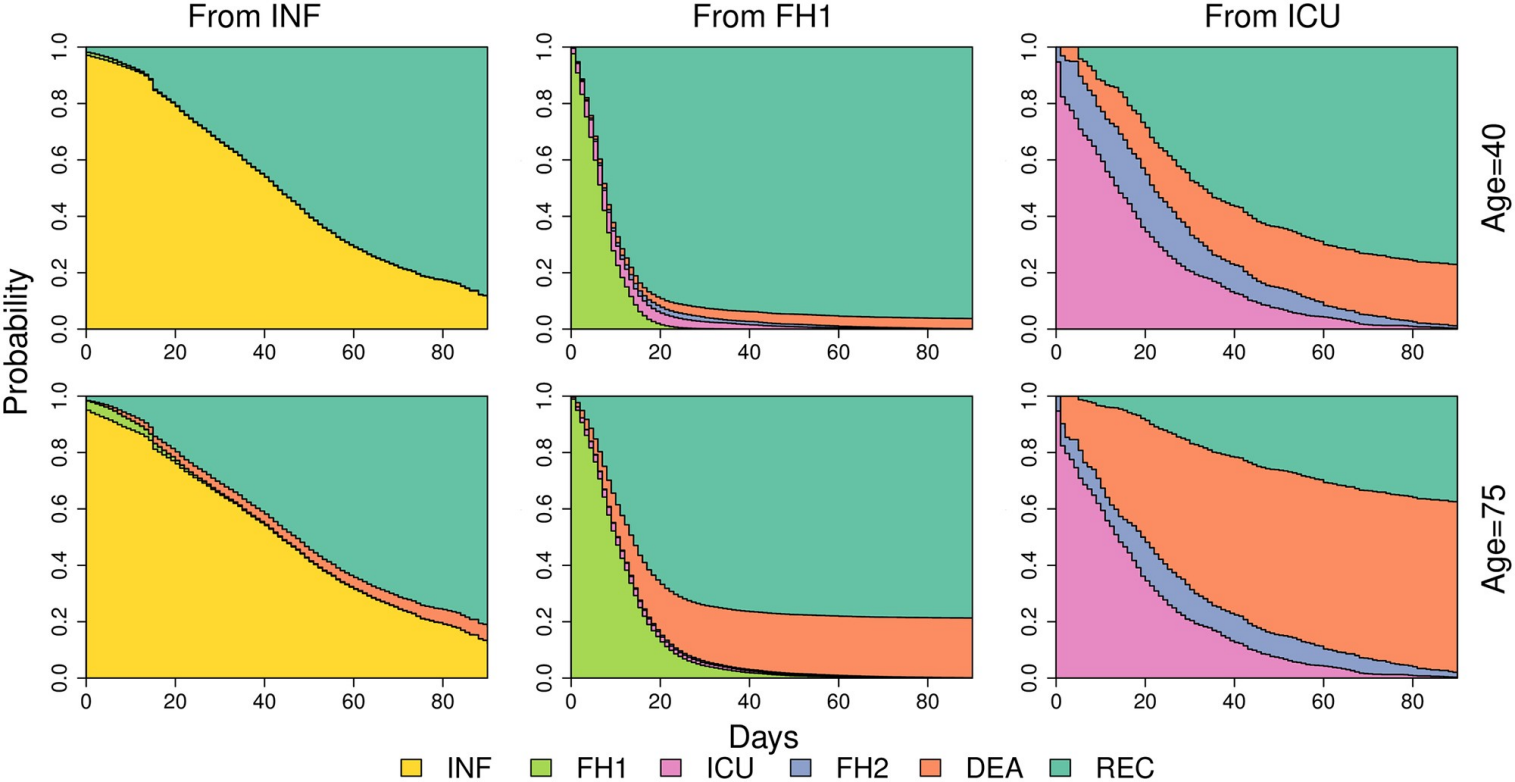
Transition probabilities for two typical patients of ages 40 and 75. The left panel shows transitions from INF to all the other states, the middle panel transitions from hospitalisation and the right panel shows transitions from ICU.

One of the more interesting features of our model is the ability to make individualised predictions. We considered two typical patients, both females with ages 40 and 75 and diagnosed at the beginning of the pandemic with no previous pathologies. Fig 3 shows the probabilities of transition to each state at the moment of diagnosis (left panel), after hospitalisation (middle panel) and after ICU (right panel). This type of representation is very useful to represent the risks associated to the current state that the patient is.

**Fig 4:**
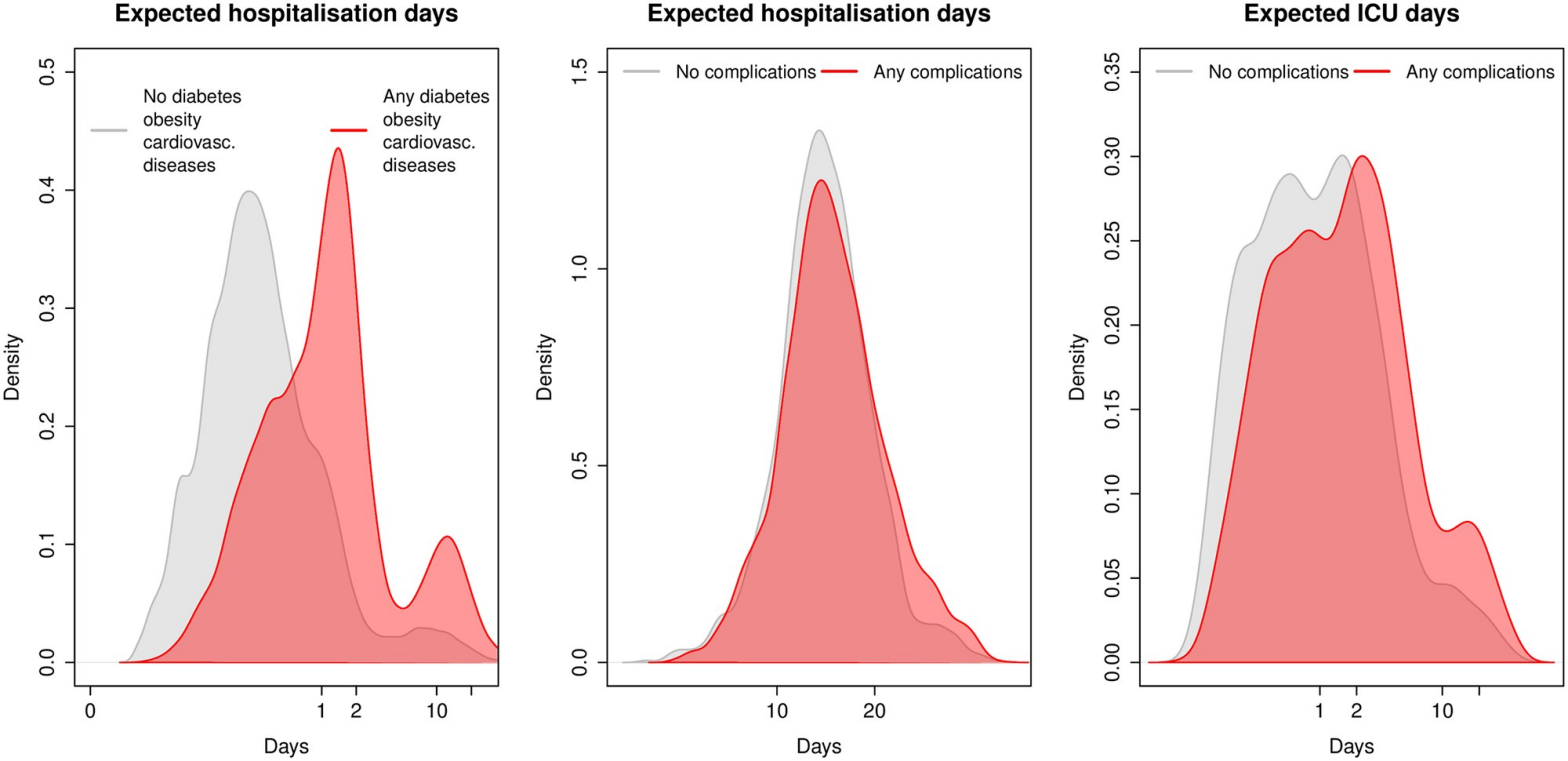
Expected length of stay distribution. The left panel shows the distribution of hospital length stays for patients with and without previous pathologies (obesity, diabetes or cardiovascular diseases). The middle panel shows the distribution of hospital length stays for patients with and without complications. The right panel shows the distribution of ICU length stays for patients with and without complications.

Based on the predictions for each patient, we computed the expected length of stay in each state. Fig 4 shows that presenting previous pathologies has a large effect in the expected length of hospitalisation, while acquired pathologies after hospitalisation will increase the length in ICU.

### Validation

**Fig 5:**
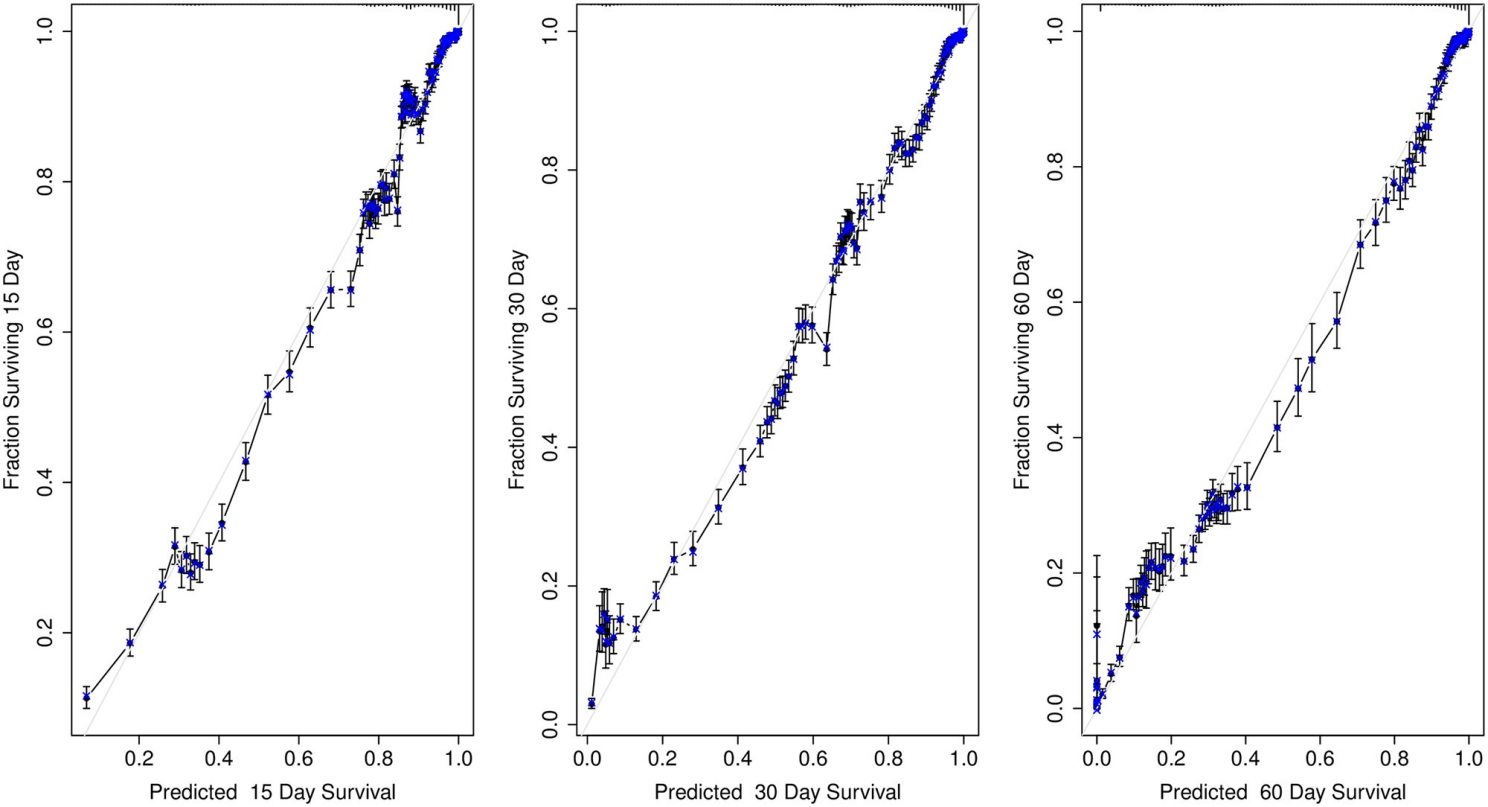
Calibration results. The plot shows internal model calibration for predictions at 15, 30 and 60 days after COVID-19 diagnosis.

In order to asses the quality of the predictions of the model, we performed several tests. First of all, we checked the proportional hazards assumption, finding that there was evidence of non-proportionality. Visual exploration of the smoothed scaled Schoenfield residual plots showed that the departure from the assumption was not severe. We performed internal validation and calibration using bootstrap [18]. Table 4 shows the optimism of the model and Fig 5 shows its calibration. These results show a stable model with a high predictive capacity.

**Table 4:** Validation indexes.

index	orig	training	test	optimism	index.corrected
		Predicted 15 days survival			
Dxy	0.7051	0.7054	0.7048	0.0005	0.7046
R2	0.1651	0.1656	0.1648	0.0008	0.1643
Slope	1.0000	1.0000	0.9967	0.0033	0.9967
D	0.0346	0.0347	0.0345	0.0002	0.0345
		Predicted 30 days survival			
Dxy	0.6962	0.6962	0.6961	0.0001	0.6961
R2	0.1651	0.1652	0.1648	0.0004	0.1648
Slope	1.0000	1.0000	0.9983	0.0017	0.9983
D	0.0346	0.0346	0.0345	0.0001	0.0345
		Predicted 60 days survival			
Dxy	0.6724	0.6728	0.6723	0.0005	0.6719
R2	0.1651	0.1654	0.1648	0.0006	0.1646
Slope	1.0000	1.0000	0.9965	0.0035	0.9965
D	0.0346	0.0347	0.0345	0.0001	0.0345

## Discussion

The study presented here describes the dynamic of patients from the moment they are detected as COVID-19 infected to the end of their clinical process, in terms of demographic, epidemiological, and clinical characteristics. Our model has two advantages over other approaches. First, it estimates overall associations between specific factors and the associated risks of different transitions, such as hospitalisation, ICU admission and death. These results can be useful for decision-makers in public health, not only for this pandemic but also for other challenges in the future; for example to elaborate a priority vaccination list that would prioritise chronic diseased patients associated with a greater risk of severity or death, considering those pathologies registered in PC records and following the strategy in this paper. Furthermore, it can also help in planning resources in hospitals. The second advantage is that the model produces individualised predictions of risk for each patient, that can be updated if the condition of the patient changes. These predictions can be very useful to monitor patients according to their needs, focusing on those patients at a higher risk of disease progression.

Many recent studies in the literature deal with predictive models for COVID-19. From a methodological point of view we should cite [20], which provides an interesting review of prediction models, and [21], that presents a concise review of common methodological errors observed in some literature dealing with predictive models during COVID-19 pandemic. This last reference puts the focus on the need to use survival models that take into account censored observations and competing risks which are often ignored in time-to-event analysis.

To our knowledge, most of the contributions on predictive models for COVID-19 progression are hampered by either a somewhat limited access to data (typically, they are exclusively based on data from hospitalised or critical patients) or by the simplistic nature of the predictive models considered (which, of course, is often related to the limited access of useful data). As an example, [4] considers a wide collection of predictors but it is based on a very small sample of hospitalised patients. This reference uses Cox regression models, but with death as the only event of interest. Access to the data provided by SACYL (the regional health service of Castilla y León) has allowed us to incorporate to our study a very important factor, which is the risk of hospitalisation of our population.

With respect to the simplistic modelling approach, we note that for most of the existing studies the set of predictive variables is limited to comorbidities or specific medications. For instance, [3] describes the progression of a moderate cohort of patients to death or discharge using survival methods but only age and gender as predictors. [5] and [6] consider large cohorts and combine data from different sources but they do not consider the ICU hospitalisation as an intermediate state. Furthermore, death is the only end event considered. [1, 2, 7–11] also consider multistate survival models with different predictors, but only with hospitalised patients and, as a consequence, cannot give any assessment of the hospitalisation risk for the general population. This comment applies also to [22], published after the submission of the first version of the present paper. This last reference focuses on the temporal evolution of risks of severe events, in the same spirit as [23, 24]. The last two papers are based on large cohorts, but the simple modeling approach used there does not allow to account, for instance, for the evolution of the length of stay in hospital or ICU. In summary, the present work is one of the very few studies that consider survival methods, censored observations, multistate and competing risk or link primary care information with hospital data. Additionally, we have also included an internal validation mechanism. In short, as far as we know, no study gives such a global vision of the dynamics of the infection like this one.

Our analysis provides severity and mortality rates estimates for the population with symptoms and hospital admitted patients stratified by different characteristics. Censored observations are taken into account by using survival methods, and unbiased mortality risk estimates are provided. The analysis gives also results relative to ICU patient’s progress, summarised as the length of stay or the factors that increase the risk of recovery and death.

Regarding risk factors associated with mortality and severity, consistent results with many other studies have been found, such as older age, being male, and different chronic diseases. Specifically, the risk of transition from FH1 to ICU increases with obesity and bilateral pneumonia but decreases with older age, reflecting the fact that admission to the ICU was restricted to older patients in the first wave. Teicoplanin has also been associated with an increased risk of entering the ICU, which can be understood as more likely to be prescribed to seriously ill patients or directly related to severity.

Our study also offers additional evidence of controversial questions as the association of Pneumococcal vaccination with an increasing risk of recovery or the Interferon treatment, which is associated with an increased risk of severity for hospitalised patients.

Our study has limitations, on the one hand, those related to the quality of the information. First, to define the cohort under study, a rigorous but not infallible criterion has been used, which implies that some patients have been incorrectly classified as COVID-19 and vice versa. Furthermore, the sample does not collect information on asymptomatic patients or patients with mild symptoms that have not been registered in the system. Moreover, as there is no systematic recording of clinical information, pharmacological prescriptions and clinical characteristics may be missing for some patients. Also, different death causes have not been taken into account.

On the other hand, as the pandemic has lasted more than one year, and this study only considers the first three months, the conclusions are limited. In particular, we have observed that the month of diagnosis is an important factor, and the violation of proportional hazards assumption suggests that the dynamics of the disease may have changed over time, as more information was obtained and the disease was better understood. However, the strategy followed in this study can be adapted to the analysis of data from longer periods. In that case, the infection time will presumably be a significant predictor as an individual effect and interacting with other predictors.

## Supporting information

S1 Table Distribution of patients by pairwise comorbidities combinations and state (including only those with frequency of at least 100 hospitalised patients). (PDF)

S2 Table Distribution of patients by pairwise drug therapies combinations and state (including only those with frequency of at least 100 hospitalised patients). (PDF)

S3 Table Coefficients and p-values for the final model obtained. (PDF)
