# Supplementary material for: Predicting COVID-19 progression from diagnosis to recovery or death linking primary care and hospital records in Castilla y León (Spain)
Source: PLoS One. 2021 Sep 20;16(9):e0257613. doi: 10.1371/journal.pone.0257613 (PMC8451995; doi:10.1371/journal.pone.0257613)
Supplement: S1 Table — (PDF) [file pone.0257613.s001.pdf]

S1 Table: Distribution of patients by pairwise comorbidities combinations and state (including only those with frequency of at least 100 hospitalised patients)

	INF		FH1		ICU		DEA		REC	
	n	%	n	%	n	%	n	%	n	%
Tobacco disorder + Cancer	766	100.0	190	24.8	15	2.0	73	9.5	541	70.6
Tobacco disorder + Psyquiatric disorder	3,784	100.0	355	9.4	31	0.8	108	2.9	2,741	72.4
Tobacco disorder + Diabetes	828	100.0	197	23.8	21	2.5	78	9.4	555	67.0
Tobacco disorder + Hypercolesterol	2,705	100.0	388	14.3	40	1.5	122	4.5	1,971	72.9
Tobacco disorder + Hypertension	1,904	100.0	379	19.9	38	2.0	130	6.8	1,358	71.3
Tobacco disorder + Hypothyroidism	708	100.0	54	7.6	3	0.4	18	2.5	533	75.3
Tobacco disorder + Cardiovascular disease	1,230	100.0	258	21.0	20	1.6	121	9.8	858	69.8
Tobacco disorder + Obesity	1,219	100.0	176	14.4	26	2.1	59	4.8	874	71.7
Tobacco disorder + Respiratory disease	4,271	100.0	498	11.7	42	1.0	162	3.8	3,098	72.5
Tobacco disorder + Neurological disease	351	100.0	63	17.9	3	0.9	27	7.7	252	71.8
Tobacco disorder + Hematological disorder	1,561	100.0	184	11.8	12	0.8	82	5.3	1,112	71.2
Tobacco disorder + Liver disease	409	100.0	80	19.6	10	2.4	24	5.9	291	71.1
Tobacco disorder + Kidney disease	381	100.0	96	25.2	7	1.8	49	12.9	252	66.1
Tobacco disorder + Other chronic disease	1,783	100.0	315	17.7	22	1.2	135	7.6	1,255	70.4
Cancer + Psyquiatric disorder	3,369	100.0	798	23.7	31	0.9	557	16.5	2,115	62.8
Cancer + Diabetes	1,354	100.0	470	34.7	21	1.6	297	21.9	819	60.5
Cancer + Hypercolesterol	2,930	100.0	786	26.8	38	1.3	468	16.0	1,848	63.1
Cancer + Hypertension	3,496	100.0	1043	29.8	43	1.2	671	19.2	2,125	60.8
Cancer + Hypothyroidism	877	100.0	164	18.7	10	1.1	111	12.7	567	64.7
Cancer + Cardiovascular disease	2,357	100.0	763	32.4	27	1.1	559	23.7	1,366	58.0
Cancer + Obesity	1,146	100.0	323	28.2	22	1.9	194	16.9	732	63.9
Cancer + Respiratory disease	4,147	100.0	1086	26.2	46	1.1	708	17.1	2,625	63.3
Cancer + Neurological disease	815	100.0	219	26.9	2	0.2	225	27.6	441	54.1
Cancer + Hematological disorder	2,094	100.0	523	25.0	19	0.9	363	17.3	1,317	62.9
Cancer + Liver disease	325	100.0	90	27.7	4	1.2	42	12.9	214	65.8
Cancer + Kidney disease	833	100.0	283	34.0	7	0.8	224	26.9	461	55.3
Cancer + Cerebrovascular disease	287	100.0	81	28.2			74	25.8	162	56.4
Cancer + Other chronic disease	2,987	100.0	891	29.8	30	1.0	627	21.0	1,793	60.0
Psyquiatric disorder + Diabetes	3,529	100.0	920	26.1	62	1.8	647	18.3	2,180	61.8
Psyquiatric disorder + Hypercolesterol	9,582	100.0	1,740	18.2	106	1.1	1,028	10.7	6,345	66.2
Psyquiatric disorder + Hypertension	9,602	100.0	2,120	22.1	103	1.1	1,470	15.3	6,051	63.0
Psyquiatric disorder + Hypothyroidism	3,632	100.0	457	12.6	23	0.6	265	7.3	2,476	68.2
Psyquiatric disorder + Cardiovascular disease	5,966	100.0	1,445	24.2	62	1.0	1,117	18.7	3,624	60.7
Psyquiatric disorder + Obesity	4,287	100.0	744	17.4	64	1.5	365	8.5	2,916	68.0
Psyquiatric disorder + Respiratory disease	15,074	100.0	2,345	15.6	122	0.8	1,480	9.8	10,020	66.5
Psyquiatric disorder + Neurological disease	3,034	100.0	662	21.8	20	0.7	618	20.4	1,744	57.5
Psyquiatric disorder + Hematological disorder	7,338	100.0	1,073	14.6	41	0.6	770	10.5	4,868	66.3
Psyquiatric disorder + Liver disease	1,137	100.0	199	17.5	18	1.6	80	7.0	783	68.9
Psyquiatric disorder + Kidney disease	2,006	100.0	520	25.9	14	0.7	430	21.4	1,187	59.2
Psyquiatric disorder + Cerebrovascular disease	791	100.0	209	26.4	9	1.1	179	22.6	440	55.6
Psyquiatric disorder + Other chronic disease	8,793	100.0	1,863	21.2	77	0.9	1,342	15.3	5,539	63.0
Diabetes + Hypercolesterol	3,882	100.0	1,084	27.9	79	2.0	639	16.5	2,471	63.7
Diabetes + Hypertension	4,724	100.0	1,348	28.5	77	1.6	866	18.3	2,922	61.9
Diabetes + Hypothyroidism	912	100.0	219	24.0	15	1.6	137	15.0	578	63.4
Diabetes + Cardiovascular disease	2,797	100.0	911	32.6	45	1.6	658	23.5	1,662	59.4
Diabetes + Obesity	2,121	100.0	565	26.6	50	2.4	292	13.8	1,386	65.3
Diabetes + Respiratory disease	4,415	100.0	1,218	27.6	71	1.6	789	17.9	2,775	62.9
Diabetes + Neurological disease	971	100.0	258	26.6	6	0.6	251	25.8	535	55.1
Diabetes + Hematological disorder	2,188	100.0	625	28.6	27	1.2	463	21.2	1,308	59.8
Diabetes + Liver disease	487	100.0	136	27.9	11	2.3	61	12.5	327	67.1
Diabetes + Kidney disease	1,160	100.0	423	36.5	16	1.4	320	27.6	654	56.4
Diabetes + Cerebrovascular disease	328	100.0	86	26.2	5	1.5	85	25.9	186	56.7
Diabetes + Other chronic disease	3,874	100.0	1190	30.7	62	1.6	829	21.4	2,352	60.7
Hypercolesterol + Hypertension	9,637	100.0	2,249	23.3	151	1.6	1,287	13.4	6,241	64.8
Hypercolesterol + Hypothyroidism	2,809	100.0	439	15.6	27	1.0	223	7.9	1,924	68.5
Hypercolesterol + Cardiovascular disease	5,312	100.0	1,422	26.8	74	1.4	965	18.2	3,299	62.1
Hypercolesterol + Obesity	3,974	100.0	831	20.9	86	2.2	380	9.6	2,709	68.2
Hypercolesterol + Respiratory disease	11,139	100.0	2,203	19.8	143	1.3	1,203	10.8	7,482	67.2
Hypercolesterol + Neurological disease	2,121	100.0	530	25.0	18	0.8	432	20.4	1,226	57.8
Hypercolesterol + Hematological disorder	4,748	100.0	951	20.0	48	1.0	607	12.8	3,099	65.3
Hypercolesterol + Liver disease	1,027	100.0	219	21.3	27	2.6	69	6.7	732	71.3
Hypercolesterol + Kidney disease	1,922	100.0	583	30.3	23	1.2	421	21.9	1,157	60.2

Continued on next page

S1 Table – continued from previous page

	INF		FH1		ICU		DEA		REC	
	n	%	n	%	n	%	n	%	n	%
Hypercolesterol + Cerebrovascular disease	646	100.0	154	23.8	7	1.1	132	20.4	358	55.4
Hypercolesterol + Other chronic disease	6,688	100.0	1,652	24.7	83	1.2	1,070	16.0	4,249	63.5
Hypertension + Hypothyroidism	2,388	100.0	488	20.4	27	1.1	297	12.4	1,524	63.8
Hypertension + Cardiovascular disease	6,891	100.0	1983	28.8	87	1.3	1,419	20.6	4,153	60.3
Hypertension + Obesity	4,741	100.0	1,064	22.4	95	2.0	523	11.0	3,176	67.0
Hypertension + Respiratory disease	11,410	100.0	2,681	23.5	145	1.3	1,704	14.9	7,258	63.6
Hypertension + Neurological disease	2,495	100.0	625	25.1	17	0.7	556	22.3	1,422	57.0
Hypertension + Hematological disorder	5,190	100.0	1,280	24.7	52	1.0	905	17.4	3,193	61.5
Hypertension + Liver disease	941	100.0	221	23.5	20	2.1	94	10.0	644	68.4
Hypertension + Kidney disease	2,677	100.0	835	31.2	26	1.0	630	23.5	1,559	58.2
Hypertension + Cerebrovascular disease	882	100.0	237	26.9	10	1.1	200	22.7	497	56.3
Hypertension + Other chronic disease	8,003	100.0	2,197	27.5	85	1.1	1,556	19.4	4,870	60.9
Hypothyroidism + Cardiovascular disease	1,481	100.0	337	22.8	17	1.1	235	15.9	947	63.9
Hypothyroidism + Obesity	1,276	100.0	210	16.5	12	0.9	89	7.0	916	71.8
Hypothyroidism + Respiratory disease	3,996	100.0	563	14.1	34	0.9	306	7.7	2,743	68.6
Hypothyroidism + Neurological disease	678	100.0	128	18.9	2	0.3	106	15.6	398	58.7
Hypothyroidism + Hematological disorder	2,433	100.0	297	12.2	15	0.6	180	7.4	1,692	69.5
Hypothyroidism + Liver disease	305	100.0	51	16.7	4	1.3	15	4.9	215	70.5
Hypothyroidism + Kidney disease	565	100.0	142	25.1	7	1.2	105	18.6	344	60.9
Hypothyroidism + Cerebrovascular disease	179	100.0	30	16.8			32	17.9	102	57.0
Hypothyroidism + Other chronic disease	2,321	100.0	419	18.1	21	0.9	267	11.5	1,556	67.0
Cardiovascular disease + Obesity	2,286	100.0	643	28.1	39	1.7	381	16.7	1,477	64.6
Cardiovascular disease + Respiratory disease	7,679	100.0	2,021	26.3	93	1.2	1,415	18.4	4,761	62.0
Cardiovascular disease + Neurological disease	1,627	100.0	436	26.8	13	0.8	434	26.7	888	54.6
Cardiovascular disease + Hematological disorder	3,643	100.0	975	26.8	26	0.7	755	20.7	2,241	61.5
Cardiovascular disease + Liver disease	560	100.0	149	26.6	11	2.0	73	13.0	371	66.2
Cardiovascular disease + Kidney disease	1,834	100.0	642	35.0	19	1.0	528	28.8	997	54.4
Cardiovascular disease + Cerebrovascular disease	627	100.0	179	28.5	10	1.6	153	24.4	349	55.7
Cardiovascular disease + Other chronic disease	6,455	100.0	1,900	29.4	68	1.1	1412	21.9	3,840	59.5
Obesity + Respiratory disease	5,345	100.0	994	18.6	94	1.8	476	8.9	3,673	68.7
Obesity + Neurological disease	723	100.0	166	23.0	9	1.2	120	16.6	459	63.5
Obesity + Hematological disorder	2,216	100.0	441	19.9	32	1.4	241	10.9	1,476	66.6
Obesity + Liver disease	535	100.0	119	22.2	16	3.0	37	6.9	384	71.8
Obesity + Kidney disease	834	100.0	254	30.5	12	1.4	184	22.1	488	58.5
Obesity + Cerebrovascular disease	228	100.0	49	21.5	3	1.3	44	19.3	134	58.8
Obesity + Other chronic disease	3,070	100.0	744	24.2	54	1.8	412	13.4	2,014	65.6
Respiratory disease + Neurological disease	2,991	100.0	676	22.6	20	0.7	601	20.1	1,769	59.1
Respiratory disease + Hematological disorder	8,379	100.0	1,350	16.1	56	0.7	932	11.1	5,530	66.0
Respiratory disease + Liver disease	1,308	100.0	272	20.8	26	2.0	103	7.9	911	69.6
Respiratory disease + Kidney disease	2,609	100.0	737	28.2	22	0.8	584	22.4	1,536	58.9
Respiratory disease + Cerebrovascular disease	875	100.0	224	25.6	9	1.0	201	23.0	499	57.0
Respiratory disease + Other chronic disease	10,793	100.0	2,383	22.1	105	1.0	1,602	14.8	6,918	64.1
Neurological disease + Hematological disorder	1,483	100.0	330	22.3	7	0.5	321	21.6	854	57.6
Neurological disease + Liver disease	187	100.0	45	24.1			23	12.3	121	64.7
Neurological disease + Kidney disease	578	100.0	162	28.0	2	0.3	157	27.2	323	55.9
Neurological disease + Cerebrovascular disease	319	100.0	79	24.8	4	1.3	82	25.7	162	50.8
Neurological disease + Other chronic disease	2,624	100.0	649	24.7	14	0.5	589	22.4	1,498	57.1
Hematological disorder + Liver disease	575	100.0	123	21.4	7	1.2	61	10.6	389	67.7
Hematological disorder + Kidney disease	1,540	100.0	443	28.8	8	0.5	373	24.2	877	56.9
Hematological disorder + Cerebrovascular disease	452	100.0	112	24.8	3	0.7	100	22.1	262	58.0
Hematological disorder + Other chronic disease	5,217	100.0	1,173	22.5	36	0.7	860	16.5	3,280	62.9
Liver disease + Kidney disease	208	100.0	62	29.8	2	1.0	35	16.8	128	61.5
Liver disease + Other chronic disease	853	100.0	219	25.7	17	2.0	97	11.4	568	66.6
Kidney disease + Cerebrovascular disease	222	100.0	76	34.2	2	0.9	70	31.5	117	52.7
Kidney disease + Other chronic disease	2,216	100.0	723	32.6	21	0.9	569	25.7	1,266	57.1
Cerebrovascular disease + Other chronic disease	822	100.0	230	28.0	10	1.2	204	24.8	462	56.2
