# Supplementary material for: Predicting COVID-19 progression from diagnosis to recovery or death linking primary care and hospital records in Castilla y León (Spain)
Source: PLoS One. 2021 Sep 20;16(9):e0257613. doi: 10.1371/journal.pone.0257613 (PMC8451995; doi:10.1371/journal.pone.0257613)
Supplement: S2 Table — (PDF) [file pone.0257613.s002.pdf]

S2 Table: Distribution of patients by pairwise drug therapies combinations and state (including only those with frequency of at least 100 hospitalised patients).

	FH1		ICU		DEA		REC	
	n	%	n	%	n	%	n	%
METHYLPREDNISOLONE + PREDNISONE	775	100.0	54	7.0	180	23.2	592	76.4
METHYLPREDNISOLONE + CEFTRIAXONE	2,155	100.0	175	8.1	709	32.9	1434	66.5
METHYLPREDNISOLONE + CEFDITOREN	283	100.0	15	5.3	64	22.6	218	77.0
METHYLPREDNISOLONE + CLARITHROMYCIN	145	100.0	14	9.7	36	24.8	109	75.2
METHYLPREDNISOLONE + AZITHROMYCIN	2,316	100.0	196	8.5	723	31.2	1577	68.1
METHYLPREDNISOLONE + LEVOFLOXACIN	1,056	100.0	75	7.1	360	34.1	687	65.1
METHYLPREDNISOLONE + LOPINAVIR AND RITONAVIR	1,348	100.0	180	13.4	379	28.1	955	70.8
METHYLPREDNISOLONE + INTERFERON BETA-1B	206	100.0	64	31.1	70	34.0	133	64.6
METHYLPREDNISOLONE + TOCILIZUMAB	330	100.0	125	37.9	79	23.9	242	73.3
METHYLPREDNISOLONE + CHLOROQUINE	207	100.0	26	12.6	58	28.0	147	71.0
METHYLPREDNISOLONE + HIDROXICHLOROQUINE	2,093	100.0	202	9.7	625	29.9	1452	69.4
PREDNISONE + CEFTRIAXONE	715	100.0	47	6.6	165	23.1	549	76.8
PREDNISONE + CEFDITOREN	210	100.0	16	7.6	42	20.0	166	79.0
PREDNISONE + CLARITHROMYCIN	165	100.0	18	10.9	34	20.6	130	78.8
PREDNISONE + AZITHROMYCIN	923	100.0	60	6.5	191	20.7	724	78.4
PREDNISONE + LEVOFLOXACIN	528	100.0	35	6.6	128	24.2	394	74.6
PREDNISONE + LOPINAVIR AND RITONAVIR	447	100.0	48	10.7	91	20.4	355	79.4
PREDNISONE + TOCILIZUMAB	161	100.0	50	31.1	20	12.4	139	86.3
PREDNISONE + HIDROXICHLOROQUINE	771	100.0	61	7.9	154	20.0	615	79.8
CEFTRIAXONE + CEFDITOREN	408	100.0	11	2.7	52	12.7	356	87.3
CEFTRIAXONE + CLARITHROMYCIN	114	100.0	12	10.5	23	20.2	91	79.8
CEFTRIAXONE + AZITHROMYCIN	3,781	100.0	263	7.0	996	26.3	2771	73.3
CEFTRIAXONE + LEVOFLOXACIN	1,071	100.0	78	7.3	368	34.4	697	65.1
CEFTRIAXONE + LOPINAVIR AND RITONAVIR	2,158	100.0	262	12.1	509	23.6	1638	75.9
CEFTRIAXONE + INTERFERON BETA-1B	288	100.0	92	31.9	96	33.3	191	66.3
CEFTRIAXONE + TOCILIZUMAB	406	100.0	150	36.9	92	22.7	306	75.4
CEFTRIAXONE + CHLOROQUINE	328	100.0	47	14.3	106	32.3	222	67.7
CEFTRIAXONE + HIDROXICHLOROQUINE	3,248	100.0	271	8.3	799	24.6	2437	75.0
CEFDITOREN + AZITHROMYCIN	463	100.0	20	4.3	76	16.4	385	83.2
CEFDITOREN + LEVOFLOXACIN	234	100.0	21	9.0	64	27.4	168	71.8
CEFDITOREN + LOPINAVIR AND RITONAVIR	208	100.0	17	8.2	34	16.3	173	83.2
CEFDITOREN + HIDROXICHLOROQUINE	394	100.0	22	5.6	63	16.0	330	83.8
CLARITHROMYCIN + AZITHROMYCIN	178	100.0	20	11.2	38	21.3	139	78.1
CLARITHROMYCIN + LEVOFLOXACIN	169	100.0	20	11.8	37	21.9	131	77.5
CLARITHROMYCIN + HIDROXICHLOROQUINE	158	100.0	19	12.0	32	20.3	126	79.7
AZITHROMYCIN + LEVOFLOXACIN	1,304	100.0	96	7.4	386	29.6	905	69.4
AZITHROMYCIN + MOXIFLOXACINO	136	100.0	11	8.1	23	16.9	112	82.4
AZITHROMYCIN + LOPINAVIR AND RITONAVIR	2,423	100.0	271	11.2	525	21.7	1885	77.8
AZITHROMYCIN + INTERFERON BETA-1B	304	100.0	102	33.6	92	30.3	210	69.1
AZITHROMYCIN + TOCILIZUMAB	512	100.0	173	33.8	109	21.3	394	77.0
AZITHROMYCIN + CHLOROQUINE	259	100.0	41	15.8	94	36.3	165	63.7
AZITHROMYCIN + HIDROXICHLOROQUINE	3,882	100.0	311	8.0	855	22.0	3011	77.6
LEVOFLOXACIN + MOXIFLOXACINO	102	100.0	11	10.8	16	15.7	85	83.3
LEVOFLOXACIN + LOPINAVIR AND RITONAVIR	771	100.0	94	12.2	252	32.7	511	66.3
LEVOFLOXACIN + INTERFERON BETA-1B	113	100.0	38	33.6	37	32.7	74	65.5
LEVOFLOXACIN + TOCILIZUMAB	161	100.0	68	42.2	36	22.4	120	74.5
LEVOFLOXACIN + HIDROXICHLOROQUINE	1,159	100.0	105	9.1	357	30.8	794	68.5
MOXIFLOXACINO + HIDROXICHLOROQUINE	109	100.0	8	7.3	21	19.3	88	80.7
LOPINAVIR AND RITONAVIR + INTERFERON BETA-1B	369	100.0	108	29.3	120	32.5	246	66.7
LOPINAVIR AND RITONAVIR + TOCILIZUMAB	487	100.0	172	35.3	120	24.6	358	73.5
LOPINAVIR AND RITONAVIR + CHLOROQUINE	348	100.0	51	14.7	91	26.1	254	73.0
LOPINAVIR AND RITONAVIR + HIDROXICHLOROQUINE	2,754	100.0	307	11.1	661	24.0	2079	75.5
INTERFERON BETA-1B + HIDROXICHLOROQUINE	393	100.0	123	31.3	131	33.3	259	65.9
TOCILIZUMAB + HIDROXICHLOROQUINE	508	100.0	179	35.2	124	24.4	375	73.8
CHLOROQUINE + HIDROXICHLOROQUINE	106	100.0	30	28.3	31	29.2	74	69.8
