# Supplementary material for: Predicting COVID-19 progression from diagnosis to recovery or death linking primary care and hospital records in Castilla y León (Spain)
Source: PLoS One. 2021 Sep 20;16(9):e0257613. doi: 10.1371/journal.pone.0257613 (PMC8451995; doi:10.1371/journal.pone.0257613)
Supplement: S3 Table — (PDF) [file pone.0257613.s003.pdf]

S3 Table: Coefficients and p-values for the final model obtained

	coef	exp(coef)	robust se	z	p
Age:INF->FH1	0.03	1.03	0.00	39.48	< 2e-16
Age:INF->DEA	0.11	1.11	0.00	48.64	< 2e-16
Age:INF->REC	-0.00	1.00	0.00	-17.16	< 2e-16
Age:FH1->ICU	-0.03	0.97	0.00	-11.86	< 2e-16
Age:FH1->DEA	0.05	1.05	0.00	20.71	< 2e-16
Age:FH1->REC	-0.02	0.98	0.00	-17.20	< 2e-16
Age:FH2->DEA	0.05	1.05	0.01	5.85	< 2e-16
Age:FH2->REC	-0.02	0.98	0.00	-3.79	< 2e-16
Female:INF->FH1	-0.41	0.67	0.02	-16.99	< 2e-16
Female:INF->DEA	-0.57	0.57	0.05	-11.84	< 2e-16
Female:FH1->ICU	-0.46	0.63	0.11	-4.28	< 2e-16
Female:FH1->REC	0.12	1.12	0.03	3.61	< 2e-16
StartApril:INF->FH1	-0.12	0.89	0.03	-4.47	< 2e-16
StartApril:INF->DEA	-0.48	0.62	0.05	-9.11	< 2e-16
StartApril:INF->REC	0.57	1.77	0.01	50.38	< 2e-16
StartApril:FH1->ICU	-1.01	0.36	0.12	-8.61	< 2e-16
StartApril:FH1->REC	0.33	1.40	0.03	9.88	< 2e-16
StartApril:ICU->FH2	0.59	1.81	0.14	4.39	< 2e-16
StartApril:FH2->REC	0.52	1.69	0.12	4.53	< 2e-16
StartMay:INF->FH1	-0.17	0.84	0.04	-4.21	< 2e-16
StartMay:INF->DEA	-1.22	0.30	0.14	-8.89	< 2e-16
StartMay:INF->REC	2.08	8.02	0.02	123.55	< 2e-16
StartMay:FH1->ICU	-1.06	0.35	0.28	-3.78	< 2e-16
StartMay:FH1->REC	0.76	2.15	0.06	12.09	< 2e-16
Tobacco-related disorders:INF->REC	0.05	1.06	0.02	3.50	< 2e-16
Cancer:INF->FH1	0.17	1.19	0.03	5.42	< 2e-16
Cancer:FH1->DEA	0.19	1.21	0.05	3.80	< 2e-16
Cancer:FH1->REC	-0.20	0.82	0.04	-4.40	< 2e-16
Psychiatric disorder:INF->DEA	0.27	1.31	0.05	5.41	< 2e-16
Diabetes:INF->FH1	0.20	1.22	0.03	6.52	< 2e-16
Diabetes:INF->DEA	0.26	1.30	0.05	4.82	< 2e-16
Hypercholesterol:INF->FH1	0.10	1.11	0.02	4.10	< 2e-16
Hypercholesterol:INF->REC	0.03	1.03	0.01	2.83	5.00e-03
Hypertension:INF->FH1	0.07	1.08	0.03	2.76	6.00e-03
Cardiovascular disease:INF->FH1	0.10	1.11	0.03	3.55	< 2e-16
Cardiovascular disease:FH1->DEA	0.20	1.22	0.05	4.23	< 2e-16
Obesity:INF->FH1	0.10	1.10	0.03	3.07	2.00e-03
Obesity:FH1->ICU	0.38	1.46	0.11	3.45	1.00e-03
Neurological disease:INF->DEA	0.47	1.60	0.06	8.32	< 2e-16
Neurological disease:INF->REC	-0.08	0.92	0.02	-3.78	< 2e-16
Neurological disease:FH1->DEA	0.25	1.28	0.06	4.47	< 2e-16
Neurological disease:FH1->REC	-0.28	0.75	0.05	-5.48	< 2e-16
Liver disease:INF->FH1	0.15	1.16	0.05	2.71	7.00e-03
Kidney disease:INF->DEA	0.20	1.22	0.06	3.18	1.00e-03
Kidney disease:FH1->REC	-0.21	0.81	0.05	-4.14	< 2e-16
Other chronic disease:INF->FH1	0.16	1.17	0.03	5.84	< 2e-16
Other chronic disease:INF->DEA	0.16	1.17	0.05	3.01	3.00e-03
Other chronic disease:FH1->REC	-0.15	0.86	0.04	-4.25	< 2e-16
Flu19 vaccine:FH1->DEA	0.21	1.23	0.05	4.26	< 2e-16
Pneumococcal vaccine:INF->FH1	0.29	1.34	0.06	4.52	< 2e-16
Pneumococcal vaccine:INF->REC	0.19	1.21	0.03	6.46	< 2e-16
Pneumococcal vaccine:FH1->REC	0.24	1.27	0.07	3.35	1.00e-03
Pneumonia:INF->FH1	2.52	12.42	0.03	94.03	< 2e-16
Pneumonia:INF->REC	-0.59	0.56	0.04	-16.46	< 2e-16
Pneumonia:FH1->REC	0.13	1.13	0.03	3.86	< 2e-16
Respiratory failure:FH1->ICU	0.63	1.87	0.15	4.11	< 2e-16
Respiratory failure:FH1->REC	-0.30	0.74	0.07	-4.28	< 2e-16
Tromboembolism event:FH1->DEA	-1.36	0.26	0.28	-4.82	< 2e-16
Septic shock:FH1->REC	-0.67	0.51	0.16	-4.08	< 2e-16
Bilateral Pneumonia:FH1->ICU	0.36	1.43	0.11	3.23	1.00e-03
Methylprednisolone:FH1->REC	-0.38	0.68	0.03	-12.11	< 2e-16
Methylprednisolone:ICU->FH2	-0.48	0.62	0.10	-4.83	< 2e-16
Methylprednisolone:FH2->REC	-0.28	0.76	0.10	-2.85	4.00e-03
Prednisone:FH1->DEA	-0.52	0.59	0.07	-7.73	< 2e-16

Continued on next page

S3 Table – continued from previous page

	coef	exp(coef)	robust se	z	p
Prednisone:FH2->DEA	-1.07	0.34	0.32	-3.32	1.00e-03
Cefditoren:FH1->DEA	-0.28	0.76	0.10	-2.77	6.00e-03
Azithromycin:FH1->DEA	-0.35	0.70	0.05	-7.61	< 2e-16
Levofloxacin:FH1->REC	-0.15	0.86	0.04	-4.02	< 2e-16
Teicoplanin:FH1->ICU	0.82	2.28	0.24	3.47	1.00e-03
Teicoplanin:FH1->REC	-1.02	0.36	0.17	-5.83	< 2e-16
Interferon beta-1b:FH1->ICU	1.01	2.74	0.13	7.61	< 2e-16
Interferon beta-1b:FH1->REC	-0.14	0.87	0.05	-2.84	5.00e-03
Interferon beta-1b:FH2->DEA	0.76	2.15	0.23	3.28	1.00e-03
Anakinra:FH1->ICU	0.69	2.00	0.23	2.96	3.00e-03
Anakinra:FH1->REC	-0.82	0.44	0.13	-6.35	< 2e-16
Tocilizumab:FH1->ICU	1.73	5.62	0.11	16.38	< 2e-16
Tocilizumab:FH1->DEA	-0.38	0.68	0.12	-3.21	1.00e-03
Tocilizumab:FH1->REC	-0.36	0.70	0.04	-8.26	< 2e-16
